# Evaluating Protein Fouling on Membranes Patterned by Woven Mesh Fabrics

**DOI:** 10.3390/membranes11100730

**Published:** 2021-09-25

**Authors:** Anna Malakian, Scott M. Husson

**Affiliations:** Department of Chemical and Biomolecular Engineering, Clemson University, Clemson, SC 29634, USA; amalaki@g.clemson.edu

**Keywords:** membrane fouling, membrane patterning, Hermia model, ultrafiltration, water purification

## Abstract

Membrane surface patterning is one approach used to mitigate fouling. This study used a combination of flux decline measurements and visualization experiments to evaluate the effectiveness of a microscale herringbone pattern for reducing protein fouling on polyvinylidene fluoride (PVDF) ultrafiltration membranes. Thermal embossing with woven mesh stamps was used for the first time to pattern membranes. Embossing process parameters were studied to identify conditions replicating the mesh patterns with high fidelity and to determine their effect on membrane permeability. Permeability increased or remained constant when patterning at low pressure (≤4.4 MPa) as a result of increased effective surface area; whereas permeability decreased at higher pressures due to surface pore-sealing of the membrane active layer upon compression. Flux decline measurements with dilute protein solutions showed monotonic decreases over time, with lower rates for patterned membranes than as-received membranes. These data were analyzed by the Hermia model to follow the transient nature of fouling. Confocal laser scanning microscopy (CLSM) provided complementary, quantitative, spatiotemporal information about protein deposition on as-received and patterned membrane surfaces. CLSM provided a greater level of detail for the early (pre-monolayer) stage of fouling than could be deduced from flux decline measurements. Images show that the protein immediately started to accumulate rapidly on the membranes, likely due to favorable hydrophobic interactions between the PVDF and protein, followed by decreasing rates of fouling with time as protein accumulated on the membrane surface. The knowledge generated in this study can be used to design membranes that inhibit fouling or otherwise direct foulants to deposit selectively in regions that minimize loss of flux.

## 1. Introduction

Membrane biofouling occurs when biomass deposits form on a membrane surface or within its pores [1]. Biofouling impairs membrane performance by increasing the mass-transfer resistance for fluid transport through the membrane, which increases operating costs [2,3,4]. Preventing it is the main objective of this and numerous other studies.

A number of factors play important roles in the fouling process and influence the fouling mechanism; for proteins, these include Coulombic and other electrostatic forces and hydrophobic interactions between protein molecules and membrane surfaces [5,6], which themselves change as fouling progresses. Correspondingly, a number of strategies can be taken to combat membrane biofouling. These include feed pretreatment steps, the adjustment of operating conditions (e.g., crossflow velocity, pH) [7], the development of new membranes [8,9], and chemical or physical modification of the membrane surfaces [10,11,12,13,14]. Modifying membrane surfaces with ordered patterns of micro- and nanoscale features can alter shear-stress profiles in ways that mitigate fouling [15,16,17]. The effects of such microscale features as shark skin mimics [18], pyramids [2,19] and line and groove patterns [20,21] on protein fouling have been studied, and results show the positive influence of patterning on decreasing the rate of fouling. Realizing these benefits through the commercial adoption of patterning will depend in part on the development of efficient, cost-effective manufacturing approaches.

Patterns can be introduced during phase inversion or post-membrane fabrication by embossing. Introducing patterns during phase inversion is normally performed by casting onto a mold. Upon solvent evaporation, the pattern features appear on the membrane surface [22]. This method avoids the added complexity of post-fabrication modification; however, a number of factors related to solution and solvent properties, wetting, and membrane-mold adhesion may cause difficulties [22,23]. Post-fabrication embossing applies a stamp under pressure to form surface patterns [24,25]. The replication of features with high fidelity can be achieved through the proper choice of embossing conditions [22]. Although patterning by micromolding is complicated, post-fabrication thermal embossing can be straightforward, using a roll-to-roll process [26]. Hutfles et al. [27] patterned ultrafiltration membranes using a roll-to-roll process at room temperature. While groundbreaking for its introduction of a roll-to-roll process for membrane patterning, the stamp was produced using a nickel master mold that required specialized equipment and was expensive to process. Thus, there is a need to develop novel patterning methods to improve manufacturability. The important innovation from our study was the identification and demonstration of woven mesh fabrics as inexpensive and widely available stamps that could be used for the commercial application of roll-to-roll patterning.

Understanding how patterned membranes become fouled is expected to inform improved designs. Oftentimes, flux decline measurements are reported to quantify the impact of fouling on membrane performance and fit to models that provide some insights on the fouling of mechanisms. Some have used atomic force microscopy (AFM) [16], electron microscopy (EM) [28,29], and confocal scanning laser microscopy (CLSM) [30] to observe, and sometimes quantify, foulant accumulation. CLSM is especially useful, as it provides information about membrane morphology and fouling over a relatively large visualization area, in the wet state, and without artifacts introduced by sample sectioning [31]. It also is capable of distinguishing multiple foulant species on the surface or co-locating the foulant and surface features. Zator et al. [32] used CLSM to characterize membrane fouling and determine cleaning efficiencies. Marroquin et al. [33] studied internal fouling of microfiltration membranes by multicomponent foulant mixtures. Of particular relevance to our study, Choi et al. [34] visualized fouling on micropatterned membranes and evaluated the role played by pattern geometry on membrane biofouling. Lee et al. [35] used CLSM to observe microbial attachment on patterned membranes. In these pioneering studies, the patterned membranes and biofilms were stained after the filtration process. In this study, we stained the membranes before filtration and used a different stain for the protein foulant to locate and quantify protein deposition on the surfaces of patterned membranes.

The aims of this study were to evaluate the efficacy of using woven mesh fabric for patterning ultrafiltration membranes and to combine flux decline measurements and visualization experiments to elucidate the mechanism(s) of protein fouling on the resulting microscale herringbone patterned membranes. Polyvinylidene fluoride (PVDF) ultrafiltration membranes were patterned by thermal embossing with polyester woven mesh for the first time. Embossing process parameters were studied to identify conditions replicating the mesh pattern with high fidelity. Flux decline data using dilute protein were collected and analyzed to uncover transient fouling mechanisms on as-received and patterned membranes. CLSM provided complementary, quantitative, spatiotemporal information about protein deposition on as-received and patterned membrane surfaces. This study contributes knowledge that is needed to inform the design of patterned membranes with features that better mitigate fouling. Additionally, demonstration of patterning using an inexpensive, widely available stamp is innovative and may be useful for commercial practice of membrane patterning in a roll-to-roll process.

## 2. Materials and Methods

### 2.1. Materials

Polyester woven mesh with 20 μm opening size (PM-E #635 polyester cloth) was purchased from Gilson Company, Inc. (Lewis Center, OH, USA). High-strength, high-temperature silicone rubber sheets were purchased from McMaster-Carr (Robbinsville, NJ, USA). PVDF ultrafiltration membranes (Synder BN; 50 kDa MWCO) and polyamide nanofiltration membranes (GE HL) were from Sterlitech (Kent, WA, USA). Bovine serum albumin (BSA, 66.4 kDa, 9048-46-8), sodium chloride (NaCl, 7647-14-5) and ethyl alcohol (anhydrous, 200 proof) were from MilliporeSigma (St. Louis, MO, USA). BSA-Alexa Fluor™ 647 conjugate, 5-(4,6-dichlorotriazinyl) aminofluorescein (5-DTAF), phosphate-buffered saline (1× PBS buffer), and sodium carbonate powder (Na_2_CO_3_, 497-19-8) were from ThermoFisher Scientific (Waltham, MA, USA).

### 2.2. Membrane Preparation

**Figure 1:**
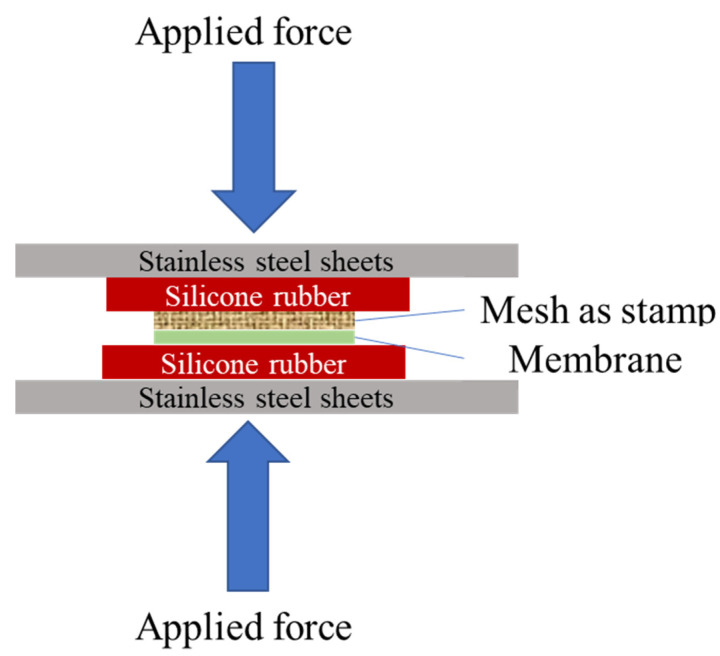
Experimental method for patterning membrane samples by thermal embossing.

Woven mesh fabric was used as the stamp to pattern 1.50 cm × 4.25 cm membrane samples by thermal embossing. Figure 1 illustrates the patterning process using a hot press (Carver, Inc. model 3851-0, Wabash, IN, USA). The temperature of hot press plates was set to 25, 45, or 65 °C. Embossing was performed for 10 min with an applied pressure that ranged from 2.66 to 17.23 MPa. The membrane was placed active side up on a silicon rubber sheet to distribute the force evenly across the sample, and the woven mesh stamp was placed on the membrane. Silicon rubber and stainless-steel sheets were placed on top of the woven mesh, and then the press plates were closed until the required pressure was reached.

The patterned and as-received PVDF membranes were labeled by 5-DTAF. First, the membrane surfaces were activated by plasma treatment (model PRC-32G, Harrick PLASMA, Ithaca, NY, USA) for 3 min at 13.33 Pa according to Singh et al. [36]. Following Marroquin et al. [31], the plasma-treated membranes were contacted with 15 µg/mL 5-DTAF in 100 mM Na_2_CO_3_ solution with 100 mM NaCl at pH 9.6 at 4 °C for 24 h to react 5-DTAF with surface hydroxyl groups. Unbound dye was removed by soaking labeled membranes for 10 min in 20% ethanol (aq) and then PBS.

### 2.3. Filtration Experiments

**Figure 2:**
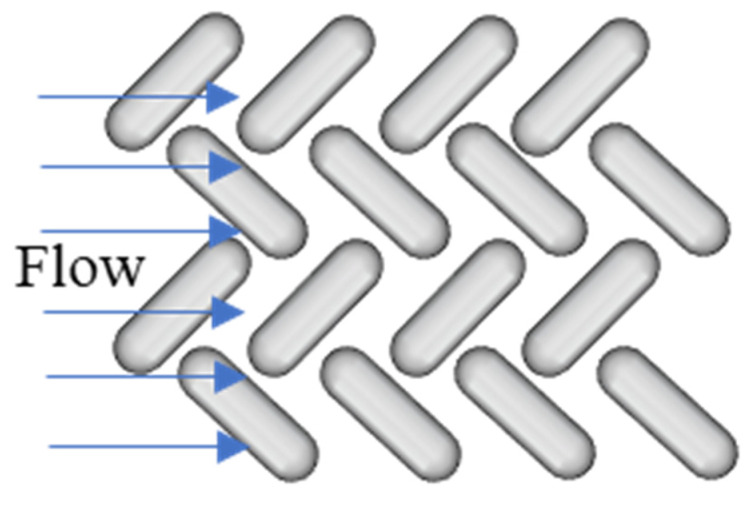
Illustration of the flow direction relative to the surface patterns.

The flux decline data were collected by a custom filtration system operated in recycle mode. Figure S1 in the Supporting Information shows the process and instrumentation diagram. Component details and vendors are provided elsewhere [15]. LabView 16 (National Instruments Corp., Austin, TX, USA) was used to program the control system and to record process data. Feed solutions were prepared by adding BSA and BSA labeled with Alexa Fluor 647 in a 20 : 1 ratio to 1× PBS buffer solution to achieve a concentration of 15 mg/L. Membranes were wetted in DI water before loading into the cell and then preconditioned by operating the system with PBS buffer for 15 min. The velocity (0.25 m/s, Re = 800) and flow direction relative to the surface patterns was kept constant for all experiments. Figure 2 shows the flow direction. Solution temperature was 23 ± 1 °C and Transmembrane Pressure (TMP) values ranged from 3.2 to 3.8 MPa to give the same initial flux of 414 ± 1 L/m^2^/h for each membrane tested. Flux data were collected every minute for patterned and as-received membranes. Samples for visualization were collected at different filtration times ranging from 10 s to 30 min. Three samples were collected for each filtration time. After filtration, all membranes were stored at −18 °C prior to visualization by confocal microscopy. BSA rejection of as-received and patterned membranes was calculated using Equation (1).

(1) $$Rejection=\frac{\left(C_{0}-C\right)}{C}\times 100$$

*C*_0_ and *C* are the concentrations of BSA in feed and permeate solutions. BSA concentrations were measured by UV spectrophotometry (U-2000, Hitachi Co., Tokyo, Japan) at a wavelength of 280 nm.

### 2.4. Membrane Characterization

#### 2.4.1. Characterization of Mesh Stamps and Patterned Membrane Surface Morphologies

A laser measuring microscope system (LEXT OLS4000 3D, Olympus Corporation, Tokyo, Japan) was used to analyze the woven mesh and the patterned membrane surfaces. Depth, width, and length of pattern features were measured using LEXT. Images were taken with a 405 nm laser source and a 20× objective lens (MPLFLN20X) with a numerical aperture of 0.45.

BET surface area, pore diameter, and pore volume of as-received and patterned membranes were measured by nitrogen adsorption–desorption isotherms using a Quantachrome Autosorb iQ Gas Sorption Analyzer (Anton-Paar, Graz, Austria). Brunauer–Emmet–Teller (BET) analysis was performed using Quantachrome^®^ ASiQwin™ software, version 5.21.

Zeta potentials were measured using a SurPASS electrokinetic analyzer (Anton-Paar GmbH, Graz, Austria) as described elsewhere [15]. Briefly, dry membranes with the active layer facing each other were mounted on a SurPASS adjustable-gap cell, washed with DI water for 1 min and then with 0.1 M potassium chloride at pH 3. The pH was increased by adding 0.1 M sodium hydroxide solution. 

Water contact angles were measured using a Krüss DSA 10-Mk2 contact angle goniometer (Krüss, Humburg, Germany). Measurements were taken 30 s after a water drop (~3 μL) was placed on the surface. They were taken at three locations on each membrane. To determine contact angle, the sessile drop model was used in DSA version 1.80.0.2 Drop Shape Analysis software.

#### 2.4.2. Visualization of Membrane Fouling by Confocal Microscopy

A Leica Microsystems (Buffalo Grove, IL, USA) SPE CLSM with an ACS Apo IMM 20× objective (numerical aperture = 0.6) was used in fluorescent mode for all fouling studies. A He-Ne laser (647 nm) and an Ar laser (488 nm) were used for excitation. The detection conditions were held constant (laser intensity of 14.99% for the He-Ne laser and 14.05% for the Ar laser, detection gain: 800, pin hole size: 94.3 μm, pixel dwell time: 1.44 μs) to allow direct comparisons among CLSM images. No digital zoom was used while taking images. CLSM was used to collect a series of lateral x–y images at different focal depths. Depending on the well depth of the patterns, a series of 10–15 images were produced by taking images from the surface of the membrane (Z = 0) to the well depth of the patterns (Z = 11 ± 3 μm). This z-series stack of images was used to create a 3-dimensional reconstruction of the membrane and to measure the mass of accumulated foulant using a calibration between fluorescence intensity and mass per unit area (vide infra).

Images were analyzed with NIH ImageJ software. Foulant was assigned a pixel intensity of zero (black), and membrane material was assigned an intensity of 255 (white). From black and white images, relative intensities of foulant on membrane surfaces were calculated.

#### 2.4.3. Calibration Curve Preparation

A calibration curve was created to measure labeled BSA surface concentrations. Solutions containing BSA Alexa Fluor 647 (in a 1:20 ratio with non-labeled BSA) were filtered through a GE HL nanofiltration membrane. Knowing the mass of protein per unit area exactly is necessary to build the calibration curve; therefore, nanofiltration membranes were used to ensure 100% protein rejection. Post-filtration, three samples from each membrane were visualized by CLSM. The average fluorescence intensity of the sample surfaces was determined by lateral x–y scans. A calibration plot was prepared relating the average fluorescence intensity to the known mass of BSA Alexa Fluor 647 per unit area of membrane.

## 3. Results and Discussion

### 3.1. Membrane Patterning

**Figure 3:**
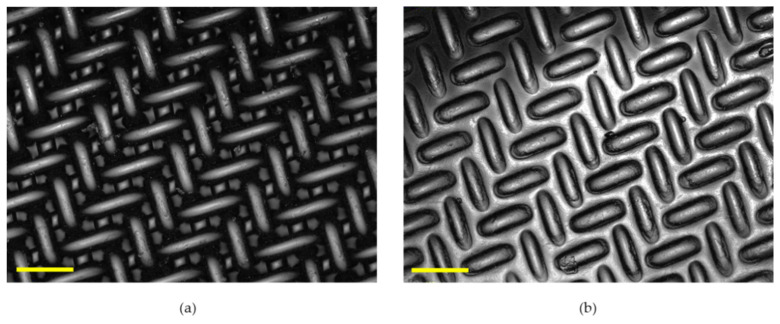
(**a**) Woven mesh and (**b**) patterned membrane images produced by LEXT using the 20× objective. The average dimensions of the mesh features are 130 ± 2 (s.d.) μm by 52 ± 3 μm with average fiber height of 30 ± 2 μm. The average end-to-end feature dimensions on the membrane are 100 ± 6 μm by 34 ± 3 μm, with average depth of 19 ± 2 μm. The common scale bar for images is 100 μm.

One aim of the study was to uncover an inexpensive stamp for patterning. We identified woven mesh fabrics as one option and discovered that patterning with the fabrics produced a herringbone pattern. Thus, we selected the stamp and the stamp determined the pattern. Ultrafiltration membranes were patterned using woven mesh stamps (Figure 3) for the first time. Embossing process parameters (T, P) were studied to identify conditions replicating the mesh patterns with high fidelity. Figure S2 in the Supporting Information shows representative LEXT images of membranes patterned at different conditions.

**Figure 4:**
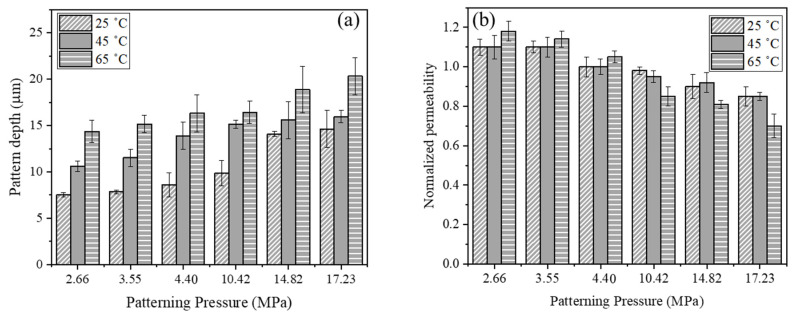
Effect of thermal embossing temperature and pressure on (**a**) average pattern feature depth and (**b**) normalized water permeability (relative to as-received membrane permeability). Feature depths were measured using LEXT with a 20× objective. Error bars represent ±1 ơ for three membrane samples.

Embossing of the polyester woven mesh was performed at three temperatures below T_g_ (80 °C) to avoid deformation of the mesh stamp, which were also well below the melting point of PVDF (160 °C), as recommended in other studies [14,24]. The experiments show that temperature is a key parameter influencing the patternability of membranes, particularly at lower embossing pressures. Figure 4a shows that average pattern feature depth increases with increasing temperature and generally increases with increasing pressure.

Pure water permeabilities were measured for the same membrane before and after patterning to assess its effect on water transport. The results in Figure 4b show that membrane permeability increased or remained constant by patterning at low pressure (≤4.4 MPa) and decreased by patterning at the higher pressures. We hypothesized that the increase in permeability at low patterning pressure results from increasing surface area following embossing; whereas, the decrease in permeability at higher pressures is likely due to surface pore-sealing of the membrane active layer due to compression. The latter part aligns with Pendergast et al. [37], who previously proposed that changes the pore size of the supports due to compaction could explain changes in permeability. At 4.4 MPa the flux was unchanged, suggesting that any membrane deformation that has occurred was compensated for by the increased surface area for transport. To test this hypothesis, we performed BET nitrogen adsorption experiments to measure the specific surface area and porosity for as-received and patterned PVDF membranes. Table 1 presents the results showing values for membranes patterned at 65 °C and 3.55 MPa, which experienced a 17% increase in permeability, and patterned at 65 °C and 17.23 MPa with a 30% permeability decrease. The results show that the nominal surface area increased by 11% after patterning, pore diameter increased 5%, and pore volume decreased 7% for low pressure patterning (3.55 MPa). These results support the first part of the hypothesis that the 17% increase in permeability for membranes patterned at low pressure (3.55 MPa) is likely attributable to increased surface area based on Darcy’s law [38] and also the increase in the membrane surface pore sizes. They also illustrate the competing effects of increased surface area and decreased pore volume. On the other hand, the 75% increase in surface area after patterning, 24% decrease in pore diameter and 65% decrease in pore volume for high pressure patterning (17.23 MPa) support the second part of hypothesis, i.e., that the 30% decrease in permeability is due to surface pore-sealing of the membrane active layer.

Maruf et al. [24] reported that using a pressure higher than the yield strength of porous PVDF membranes produced optimum patterning on the membrane surface. To test whether this principle applies to patterning by polymeric mesh stamps, we measured the yield strength of the PVDF membranes from stress–strain curves [24] collected at a 0.01 s^−1^ strain rate. Table 2 summarizes the yield strength values. Figure 4a shows no clear evidence of improved patternability by changing the pressure from just below the yield strength to just above it. However, Figure 4b shows that membrane permeability begins to decrease significantly as the patterning pressure approaches and exceeds the yield strength. This finding provides additional evidence that surface pore sealing contributes to the loss of membrane permeability at higher embossing pressures.

The stability of patterns was studied by LEXT imaging of membrane surfaces before and after 2 h of pure water filtration. Feature stability depends on irreversible deformation. Figure S3c shows that the average pattern feature depth decreased by 20–30%. This finding is consistent with the results from Idarraga-Mora et al. [39], who studied the deformation of nanocomposite membrane supports upon compression. They observed that only ~20% of the initial deformation was reversible for Matrimid supports that were subjected to a compressive stress of 1.2 MPa. We attribute the 20–30% decrease in feature depth to this reversible contribution to deformation.

**Figure 5:**
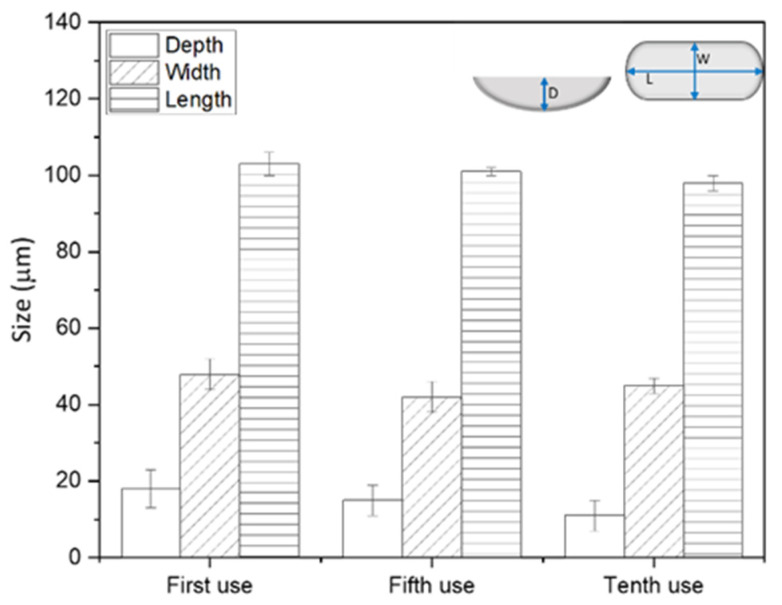
Average pattern feature dimensions for membranes that were patterned with the same mesh stamp up to 10 times at 65 °C and 3.55 MPa. Measurements were made using LEXT with a 20× objective. Error bars represent ±1 ơ from measurements on five pattern features.

Finally, measurements were performed to assess the robustness and reusability of woven mesh stamps, as reuse would be required for roll-to-roll processing [27]. Figure 5 shows average pattern feature dimensions for membranes that were patterned up to 10 times at 65 °C and 3.55 MPa with the same mesh stamp. The average values were calculated from LEXT images of the patterned membranes (Figure S4) and paired *t*-tests were conducted to compare the feature dimensions of the patterned membranes after the first and tenth use. The results of the statistical tests are given in Table S1. The average values for depth, width, and length of pattern features were statistically the same at 95% confidence, which suggests that the polyester woven mesh can be reused as a stamp.

### 3.2. Flux Decline Measurements

**Figure 6:**
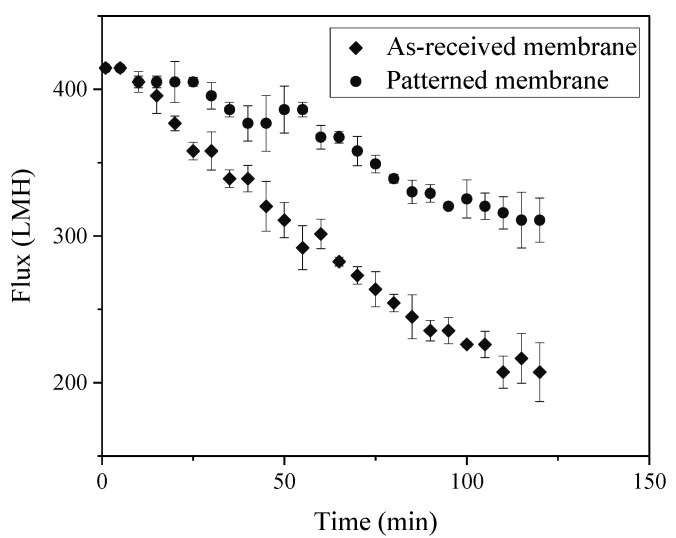
Bovine serum albumin (BSA) fouling tests. The initial flux was 414 ± 1 LMH for both samples. Error bars represent ±1 ơ among three samples.

Membranes for BSA filtration studies were patterned at 65 °C and 3.55 MPa, which produced membranes with high pattern fidelity and enhanced permeability. The BSA rejection increased from 96.2 ± 0.8 (s.d.) to 98.2 ± 1.0%; however, this increase is not statistically significant at 95% confidence. Figure 6 shows flux versus time data collected for filtration of 15 mg/L BSA solution by as-received and patterned membranes. The starting pressures were adjusted to produce the same initial permeate flux for all experiments. This procedure allows direct comparison of results because it ensures that the initial rate of foulant transport to the surface is the same. The flux declined monotonically, with a lower rate for patterned membranes. We performed a model-based analysis of the data to postulate the most likely fouling mechanism(s). We theorize that such an analysis of the flux decline data combined with CLSM visualization data can improve our understanding of how membrane patterning influences fouling.

One approach is to analyze these data is to replot them on a logarithmic scale of *d*^2^*t*/*dV*^2^ versus *dt*/*dV*, where *V* is the cumulative permeate volume. Field et al. [40] revised this Hermia model approach for crossflow filtration by including a term that considers foulant removal.

(2) $$\frac{d^{2}t}{dV^{2}}=k{\left(\frac{dt}{dV}\right)}^{n}$$

In Equation (2), parameter *n* depends on the fouling mechanism. Figure S5a in Supporting Information was used to determine that *n* = 1.0 (intermediate pore blocking) for the patterned membrane over the entire 2 h filtration run. In Figure S5b for as-received membranes, we observed a linear relationship with *n* > 2.0 in the early stage of filtration (first 20 min), suggesting that initial protein deposition did not block or seal pores [33]. Thereafter, the slope reached *n* = 2.0, suggesting a complete pore blocking mechanism. After 35 min of filtration, the slope changed to *n* = 1.1 for a short duration, indicating an intermediate pore blocking mechanism. At later times, *d*^2^*t*/*dV*^2^ reached a maximum. Ho and Zydney [41] indicate that the maximum occurs when more than 90% of the membrane surface is covered by protein aggregates, after which cake filtration becomes the dominate mechanism and the *d*^2^*t*/*dV*^2^ value decays to a constant value (*n* = 0). Before reaching the constant value, the negative slope on the plot reflects the transition between pore blocking and cake filtration where a large reduction occurs in the flux decline rate [41]. The results of the flux decline measurements and data analysis are consistent with numerous other studies that have shown how patterning with micron-scale features can reduce fouling [17,42,43]. Some of the reasons for the decreased fouling propensity include increased shear stress on the apex regions of patterned membranes, vortex formation in regions of the pattern valleys, and selective particle accumulation in the valleys during the initial stage of fouling [15,35,43,44].

### 3.3. Visualization of Membrane Fouling by CLSM

CLSM was applied to visualize and characterize fouling, especially early stage fouling where interactions between the foulant and membrane can be studied. Qualitative and quantitative spatiotemporal data on foulant accumulation were obtained by CLSM. For quantitative data, the intensity of images was measured by ImageJ software. Figure S6 presents the calibration curve. The areal mass of BSA on the membrane surfaces at different filtration times was determined by applying the calibration curve to the intensity profile. Previous studies have combined flux decline measurements with quantitative visual analysis of CLSM images to better understand protein fouling [45]. Here, we used this combined method to study the location and extent of BSA fouling on membranes patterned with a herringbone geometry. Such knowledge can be used to design membranes that inhibit fouling or otherwise direct foulants to deposit selectively in regions that minimize loss of flux.

#### 3.3.1. CLSM Analysis of Early-Stage Fouling

**Figure 7:**
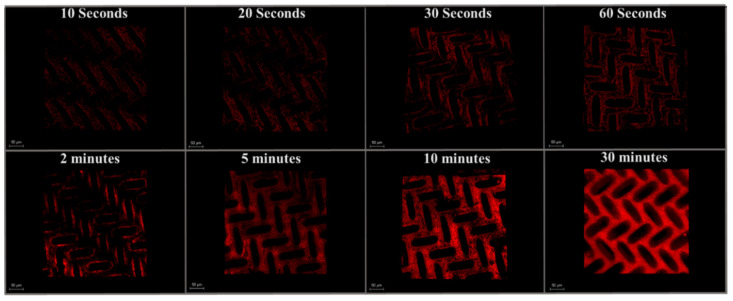
Fouling profile images of (BSA) at eight filtration times on the surface of a patterned membrane. Scale bar is 50 μm.

**Figure 8:**
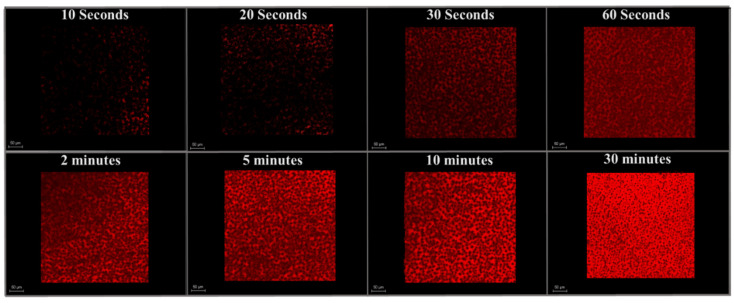
Fouling profile images of BSA at eight filtration times on the surface of an as-received membrane. Scale bar is 50 μm.

Membranes and BSA were labeled with different fluorophores. Therefore, we were able to use CLSM to image the membrane surface (green) and BSA (red) simultaneously. Figure 7 and Figure 8 show the fouling profile images of BSA at eight different filtration times on patterned and as-received membranes. Images are presented as overlay compilations of lateral x–y images taken at depth increments of 1.23 μm from the membrane surface (Z = 0) to the bottom of the pattern (Z = 11 ± 3 μm). Compiling these images was necessary to enable the direct comparison of measured intensities (areal protein masses) for as-received and patterned membranes.

**Figure 9:**
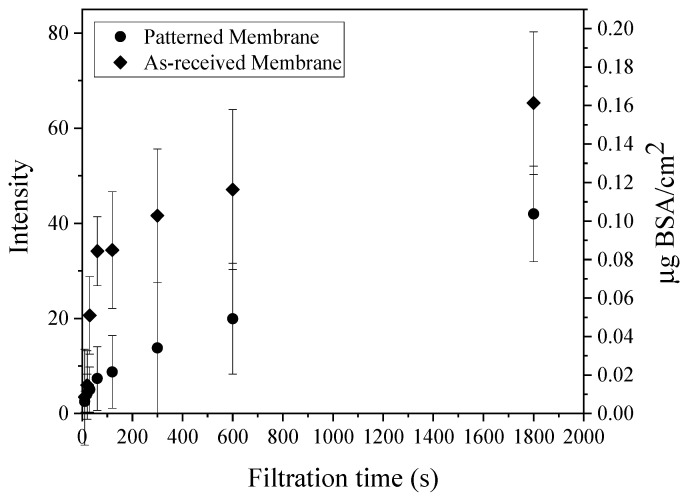
Intensity and areal mass values for confocal laser scanning microscopy (CLSM) images presented in Figure 7 and Figure 8. Each point represents a different membrane sample. Error bars represent ±1 ơ on three images from each sample.

Figure 9 shows quantitative analysis of the CLSM images shown in Figure 7 and Figure 8 for as-received and patterned membranes at different filtration times. These images provide complimentary insights to the flux decline data on fouling mechanism. Specifically, they show that the protein immediately starts to accumulate rapidly on the membranes at pH 7, due to favorable hydrophobic interactions between the PVDF and BSA [6,46], as well as the presence of positively charged domains (lysine, histidine) on the BSA surface [47]—the membrane surface is charged negative at pH 7.0 before and after staining, and the membrane surface is hydrophobic before and after staining (Table 3). The rate of fouling is highest at the onset of filtration and then decreases with time as BSA accumulates on the membrane surface, which remains negatively charged [48]. This transient fouling rate is consistent with previous studies. Rodenhausen et al. [49] found that the initial rate is highest as BSA is in contact with excess sorption sites and then decreases as binding sites are occupied by adsorbed BSA. Phan et al. [47] studied BSA adsorption on self-assembled monolayer surfaces. Using spectroscopic ellipsometry, they measured the equilibrium BSA uptake to be 0.23 μg/cm^2^ on a negatively charged, hydrophilic surface and 0.10 μg/cm^2^ on a neutral, hydrophobic surface at pH 6.7. Based on the approximate dimensions of BSA in aqueous solution, they concluded that BSA was arranged as a monolayer on these surfaces. Assuming monolayer coverage on our negatively charged, hydrophobic surface to be between 0.10 and 0.23 μg/cm^2^, our intensity measurements suggest that it takes from about 3 to 70 min to form a monolayer of BSA on the as-received membrane and from about 30 to 90 min on the patterned membrane.

As discussed earlier, Ho and Zydney [41] showed that cake layer formation begins when approximately 90% of the surface is covered with protein aggregates comprising chains of BSA molecules formed by intermolecular disulfide bonds [50]. Applying their finding to the current set of flux decline data, it appears that the as-received membrane surface is mostly covered by BSA aggregates after 90 min (i.e., where *d*^2^*t*/*dV*^2^ reached a maximum). While this finding is slightly different from the CLSM intensity measurements that showed monolayer coverage from 3 to 70 min, a primary difference between these characterization methods is that CLSM visualizes and quantifies the accumulation of proteins (including protein monomers) on the membrane surface; whereas model-based analysis of flux decline data is based on pore blocking (largely by aggregates). Previous studies [51] have shown that the fouling occurs on more than just the membrane pores. Thus, while the Hermia model can provide some information about the fouling mechanism(s) using simple mathematical expressions [52], it does not give details about the onset of fouling when pore blocking is insignificant.

Models that describe fouling often assume that a single mechanism prevails throughout the entire duration of filtration [53] or fail to describe early stages of fouling. For example, as discussed above, the Hermia model provides no mechanistic insights into the early stages of filtration [33], for example where steep slopes (*n* > 2) are observed, such as those depicted in Figure S5. Such models serve as a useful tool to assess flux decline data for further mechanistic studies; however, complementary tools are needed to understand the early stages of fouling. Using CLSM imaging in this study provided a greater level of detail for the early stage (pre-monolayer) fouling than could be deduced from flux decline measurements; it revealed high rates of initial fouling due to protein adsorption and demonstrated that this rate was different for as-received and patterned membranes. Given that the test conditions were the same, it appears that differences in hydrodynamics at the two membrane surfaces influenced the rate of attainment of BSA monolayer coverage and delayed the onset of pore blocking.

#### 3.3.2. Visualizing Spatial Deposition of Foulant

**Figure 10:**
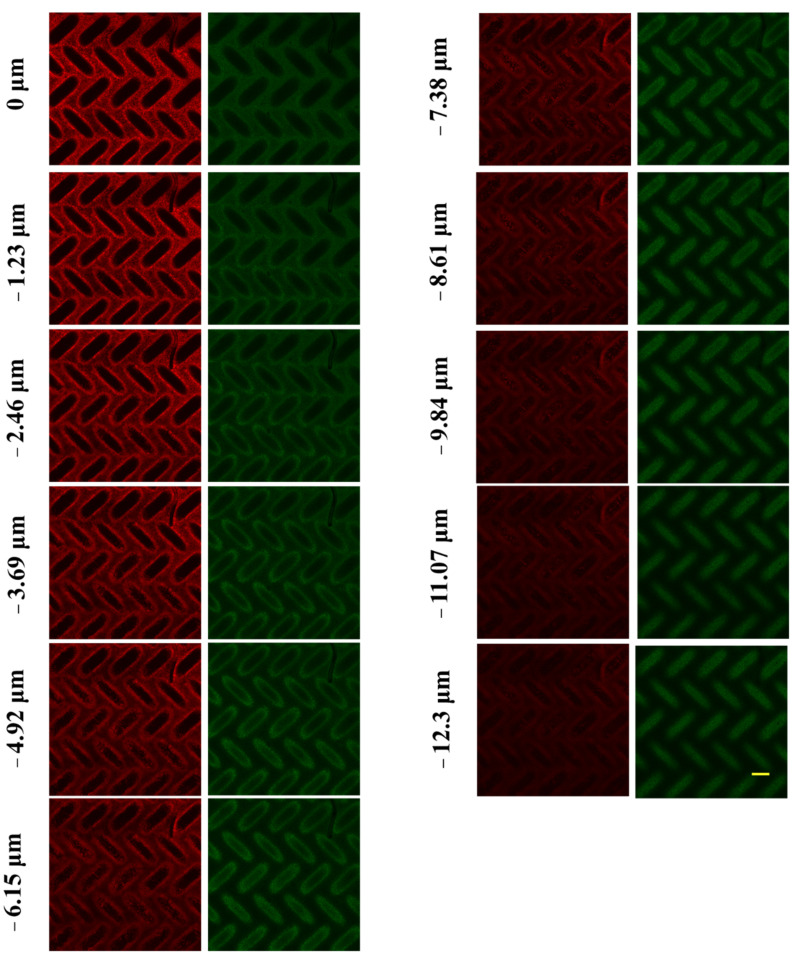
Confocal images after 2 min filtration of BSA solution. Images are shown for patterned membrane (green) and BSA foulant (red) at different depths from the surface (Z = 0). For better observation by the reader, light corrections (Brightness: +40% and Contrast: −40%) were applied to the images. The common scale bar is 50 μm.

CLSM also provided qualitative information on the location of BSA deposition during early stages of fouling. Figure 10 shows lateral x–y stacks for 2 min filtration at different depths from the membrane surface. (Figures S7–S13 show the fouling profiles for other filtration times.) The accumulation of BSA is greatest at the membrane surface for all filtration times. By embossing the membrane surface with recessive features, shear stress can be expected to increase within the recessions by creation of local vortices [28,34,54]. On such a surface, bulk and vortex streamlines develop, and the velocity profile in the recessions will depend strongly on the crossflow velocity [35]. Choi et al. [28] observed in their CFD study that for small particles such as BSA (dimension: 140 × 40 × 40 Å), moving from bulk to vortex streams is difficult, resulting in a reduction of deposited particles inside the recessions. We believe this is the cause for the low degree of BSA fouling observed within the pattern recessions. Won et al. [2] studied the fouling of membranes with prism patterns and observed that the vortex streams that developed in the valley between pattern features enabled particles to escape from the valley back to the bulk stream. In the absence of a vortex stream or where it was diminished, the particles deposited in the valley due to permeation drag. For mitigating particle deposition in the valley regions, they developed new patterns by introducing flat interval regions (400 and 800 μm) between prism features. These regions enhanced the vortex stream and reduced particle deposition. Based on their study, we theorize that the long (100 μm) valley regions of our hemi-ellipsoid pattern structure further limit the amount of protein fouling within these regions. Work is underway to understand how the herringbone pattern of the hemi-ellipsoid recessions influences vortex development and shear stress profiles inside the recessions and at the surface.

## 4. Conclusions

This study introduced a new membrane surface patterning strategy using woven mesh fabric stamps that is capable of patterning large membrane areas while retaining or even increasing membrane permeability. Increased permeability at low patterning pressures was found to correlate with increased surface area. This study also validated the reusability of the woven mesh stamps, which is important for their use in continuous roll-to-roll patterning. Flux decline measurements and visualization experiments using confocal laser scanning microscopy provided the spatiotemporal information that was needed to understand the fouling behavior of the protein solutions on the as-received and patterned membranes. This combined approach provided insights into the fouling mechanisms from the earliest stages of fouling, dominated by protein adsorption, to later stages, dominated by cake layer formation; and revealed differences between the membranes. Details of the early (pre-monolayer) stage of fouling provided by microscopy show that the protein immediately started to accumulate rapidly on the membranes due to favorable hydrophobic interactions between the PVDF and protein, while the flux decline measurements show the decreasing rates of fouling with time as proteins accumulated on the membrane surface. The results demonstrate that introduction of a herringbone pattern on the surface of PVDF ultrafiltration membranes significantly reduces protein fouling relative to as-received membranes. Extending the approach to other patterns and multicomponent solutions is expected to inform surface modification strategies used for the control of biofouling.

## Figures and Tables

**Table 1:** Brunauer–Emmet–Teller (BET) analysis data.

Membranes	BET Surface Area (m^2^/g)	Pore Diameter (nm)	Pore Volume (cc/g)
As-received Membrane	5.76	20.1	7.79 × 10^−3^
Patterned Membrane 65 °C, 3.55 MPa	6.43	21.2	7.21 × 10^−3^
Patterned Membrane 65 °C, 17.23 MPa	10.13	15.1	2.75 × 10^−3^

**Table 2:** Offset yield strength of the polyvinylidene fluoride (PVDF) membranes. Each value represents the mean calculated from measurements on three samples, and the errors represent ±1 ơ.

Temperature (°C)	Yield Strength (MPa)
25	5.11 ± 0.12
45	5.01 ± 0.24
65	4.39 ± 0.44

**Table 3:** Membranes surface characteristics. Each value represents the mean calculated from three measurements per sample, and the errors represent ±1 s.

Membranes	Contact Angle (°)	Zeta Potential (mV)
As-received Membrane	98 ± 5	−40 ± 3
Stained Membrane	85 ± 3	−80 ± 9
Patterned Membranes	89 ± 1	-

## Data Availability

The data presented in this study are available on request from the corresponding author.

## Supplementary Materials

The following are available online at https://www.mdpi.com/article/10.3390/membranes11100730/s1, Figure S1: Apparatus for flux measurements; Figure S2: LEXT images of membranes patterned at different conditions; Figure S3: LEXT images of membranes before and after 2 h filtration; Figure S4: LEXT images of membranes repeatedly patterned with same mesh stamp; Figure S5: Flux decline analysis for BSA filtration with patterned and as-received membranes, Figure S6: Calibration data relating fluorescence intensity to areal mass for BSA-Alexa Fluor™ 647 conjugate; Figure S7: 3D orthogonal reconstruction of CLSM images for patterned membrane after 10 s filtration of BSA solution; Figure S8: 3D orthogonal reconstruction of CLSM images for patterned membrane after 20 s filtration of BSA solution; Figure S9: 3D orthogonal reconstruction of CLSM images for patterned membrane after 30 s filtration of BSA solution; Figure S10: 3D orthogonal reconstruction of CLSM images for patterned membrane after 60 s filtration of BSA solution; Figure S11: 3D orthogonal reconstruction of CLSM images for patterned membrane after 5 min filtration of BSA solution; Figure S12: 3D orthogonal reconstruction of CLSM images for patterned membrane after 10 min filtration of BSA solution; Figure S13: 3D orthogonal reconstruction of CLSM images for patterned membrane after 30 min filtration of BSA solution; Table S1: Results of paired *t*-tests on membrane pattern feature sizes.
