# Supplementary material for: Evaluating Protein Fouling on Membranes Patterned by Woven Mesh Fabrics
Source: Membranes (Basel). 2021 Sep 25;11(10):730. doi: 10.3390/membranes11100730 (PMC8538970; doi:10.3390/membranes11100730)
Supplement: Supplementary file 1 [file membranes-11-00730-s001.zip › membranes-1376480-supplementary.pdf]

membranes

Supplementary Materials

Evaluating Protein Fouling on Membranes Patterned by Woven Mesh Fabrics

Anna Malakian and Scott M. Husson *

Department of Chemical and Biomolecular Engineering, Clemson University, Clemson, SC 29634, USA; amalaki@g.clemson.edu
* Correspondence: shusson@clemson.edu; Tel: +1-(864)-656-4502; Fax: +1-(864)-656-0784

Citation: Malakian, A.; Husson, S.M. Evaluating Protein Fouling on Membranes Patterned by Woven Mesh Fabrics. *Membranes* **2021**, *11*, 730. https://doi.org/10.3390/membranes11100730

Academic Editor: Gaetano Di Bella

Received: 26 August 2021
Accepted: 22 September 2021
Published: 25 September 2021

Publisher's Note: MDPI stays neutral with regard to jurisdictional claims in published maps and institutional affiliations.

Copyright: © 2021 by the authors. Licensee MDPI, Basel, Switzerland. This article is an open access article distributed under the terms and conditions of the Creative Commons Attribution (CC BY) license (http://creativecommons.org/licenses/by/4.0/).

Content

Figure S1: Apparatus for flux measurements
Figure S2: LEXT images of membranes patterned at different conditions
Figure S3: LEXT images of membranes before and after 2 h filtration
Figure S4: LEXT images of membranes repeatedly patterned with same mesh stamp
Figure S5: Flux decline analysis for BSA filtration with patterned and as-received membranes
Figure S6: Calibration data relating fluorescence intensity to areal mass for BSA-Alexa Fluor™ 647 conjugate
Figure S7: 3D orthogonal reconstruction of CLSM images for patterned membrane after 10 s filtration of BSA solution
Figure S8: 3D orthogonal reconstruction of CLSM images for patterned membrane after 20 s filtration of BSA solution
Figure S9: 3D orthogonal reconstruction of CLSM images for patterned membrane after 30 s filtration of BSA solution
Figure S10: 3D orthogonal reconstruction of CLSM images for patterned membrane after 60 s filtration of BSA solution
Figure S11: 3D orthogonal reconstruction of CLSM images for patterned membrane after 5 min filtration of BSA solution
Figure S12: 3D orthogonal reconstruction of CLSM images for patterned membrane after 10 min filtration of BSA solution
Figure S13: 3D orthogonal reconstruction of CLSM images for patterned membrane after 30 min filtration of BSA solution
Table S1: Results of paired t-tests on membrane pattern feature sizes

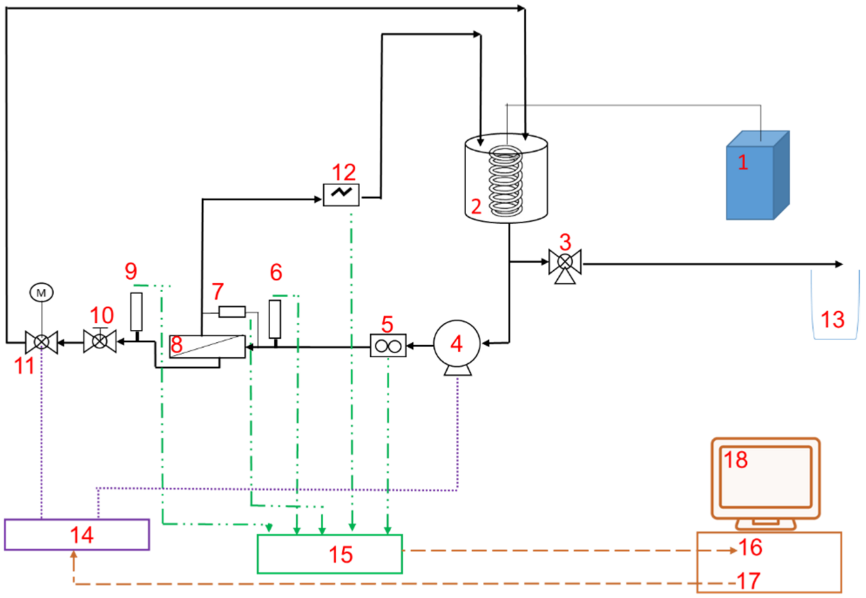

Figure S1. Apparatus for flux measurements. The system is designed to make constant-flux measurements. System components: Feed tank (1), Chiller unit (2), 3-way valve (3), Feed micropump (4), Feed volumetric flow meter (5), Feed pressure transducer (6), Differential pressure transducer (7), Membrane cell (8), Retentate pressure transducer (9), Screw-down needle valve (10), Electrical valve control (11), Permeate mass flow meter (12), Drain (13), Connector blocks(14 and 15), Analog output PCI device (16), Analog input PCI device (17), Computer with LabView operating software (18).

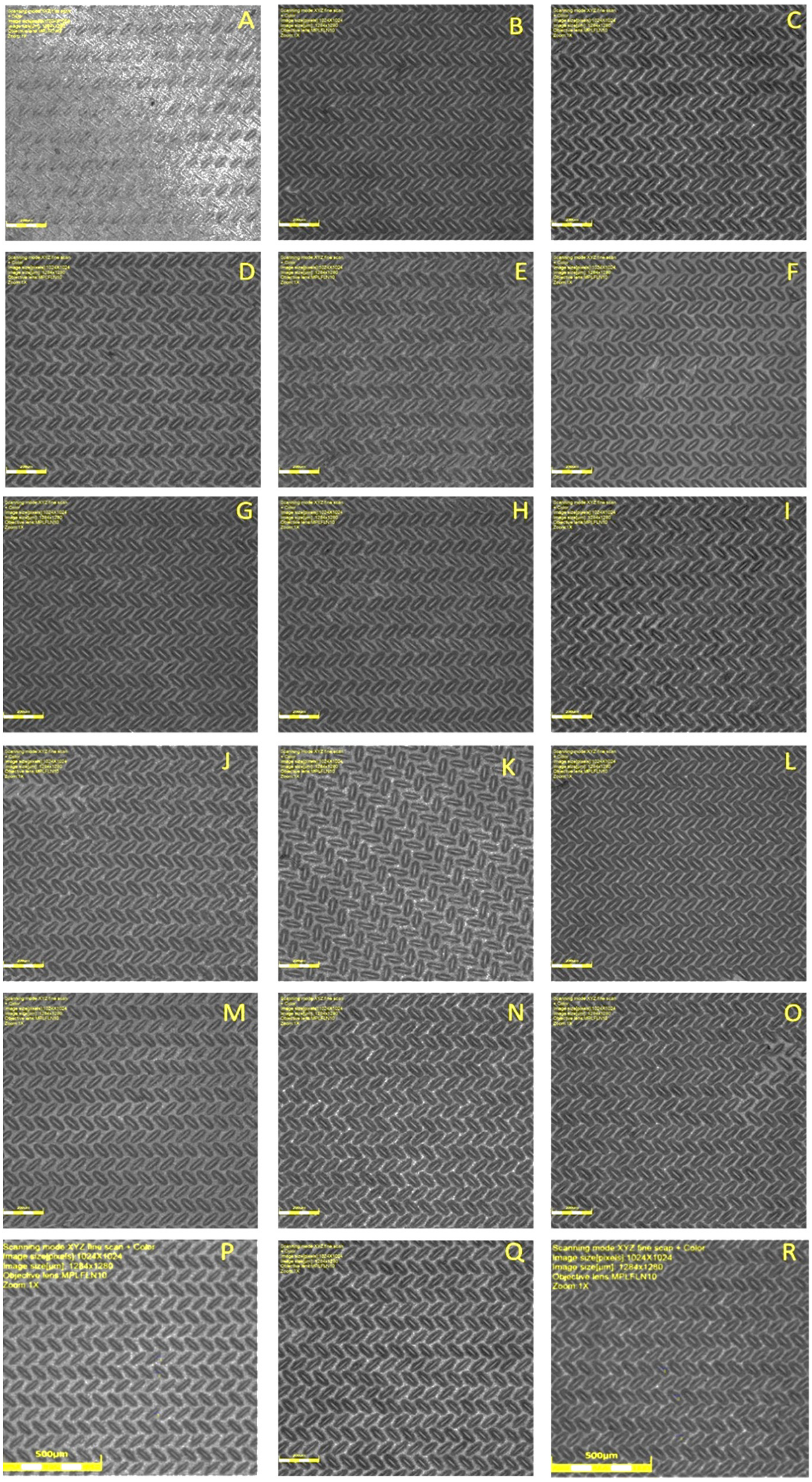

Figure S2. LEXT images of membranes patterned at different conditions. (**A**) T= 25˚C, P= 2.66 MPa; (**B**) T= 45˚C, P= 2.66 MPa; (**C**) T= 65 °C, P= 2.66 MPa; (**D**) T= 25 °C, P= 3.55 MPa; (**E**) T= 45 °C, P= 3.55 MPa; (**F**) T= 65 °C, P= 3.55 MPa; (**G**) T= 25 °C, P= 4.4 MPa; (**H**) T= 45 °C, P= 4.4 MPa; (**I**) T= 65 °C, P= 4.4 MPa; (**J**) T= 25 °C, P= 10.42 MPa; (**K**) T= 45 °C, P= 10.42 MPa; (**L**) T= 65 °C, P= 10.42 MPa; (**M**) T= 25 °C, P= 14.82 MPa; (**N**) T= 45 °C, P= 14.82 MPa; (**O**) T= 65 °C, P= 14.82 MPa; (**P**) T= 25 °C, P= 17.23 MPa; (**Q**) T= 45 °C, P= 17.23 MPa; (**R**) T= 65 °C, P= 17.23 MPa. The scale bar in images A-O and Q is 200 μm and in P and R is 500 μm.

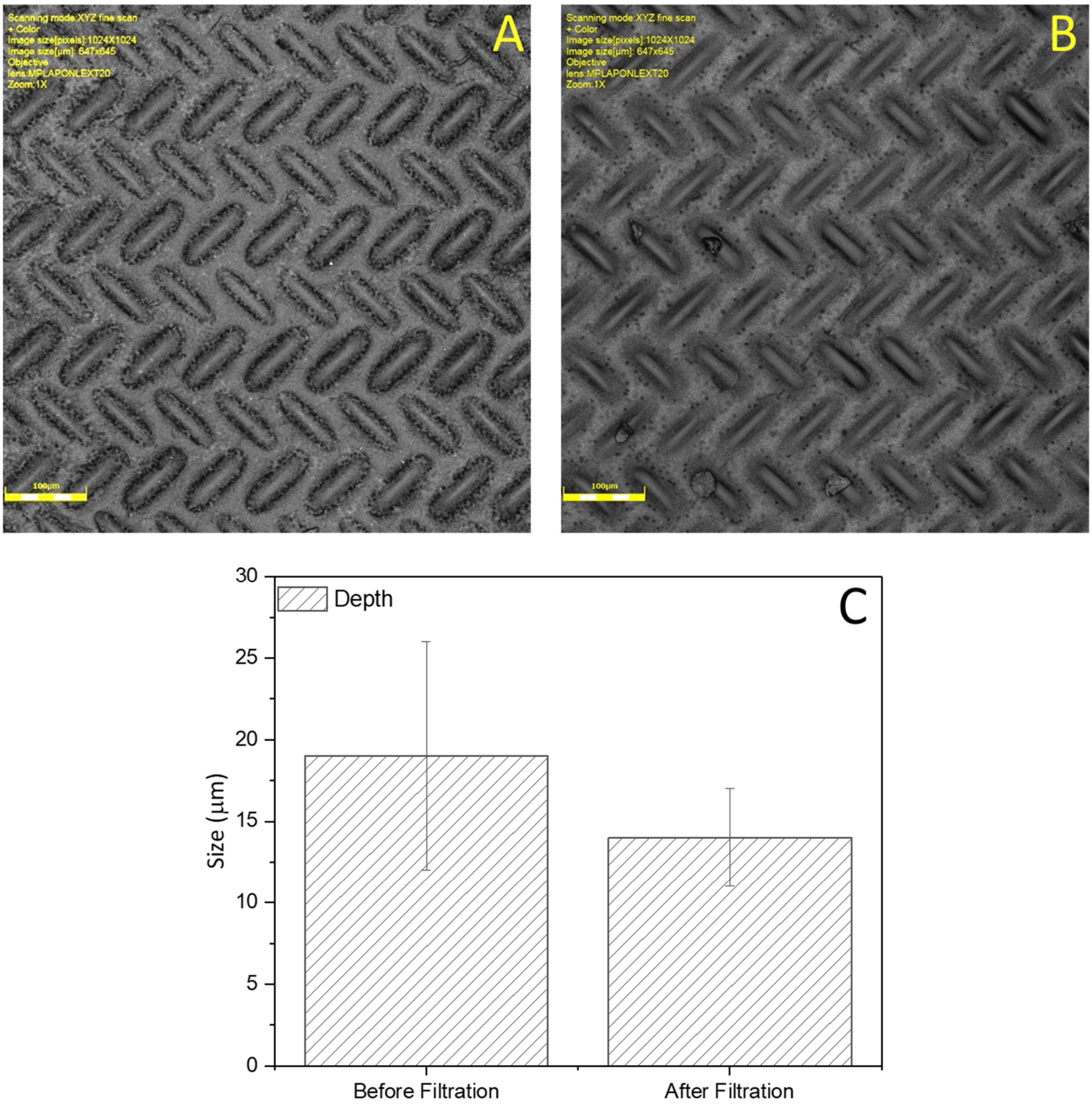

Figure S3. LEXT images of patterned membranes (**A**) before filtration, (**B**) after 2 h of pure water filtration, (**C**) average pattern feature depth for patterned membrane before and after filtration.

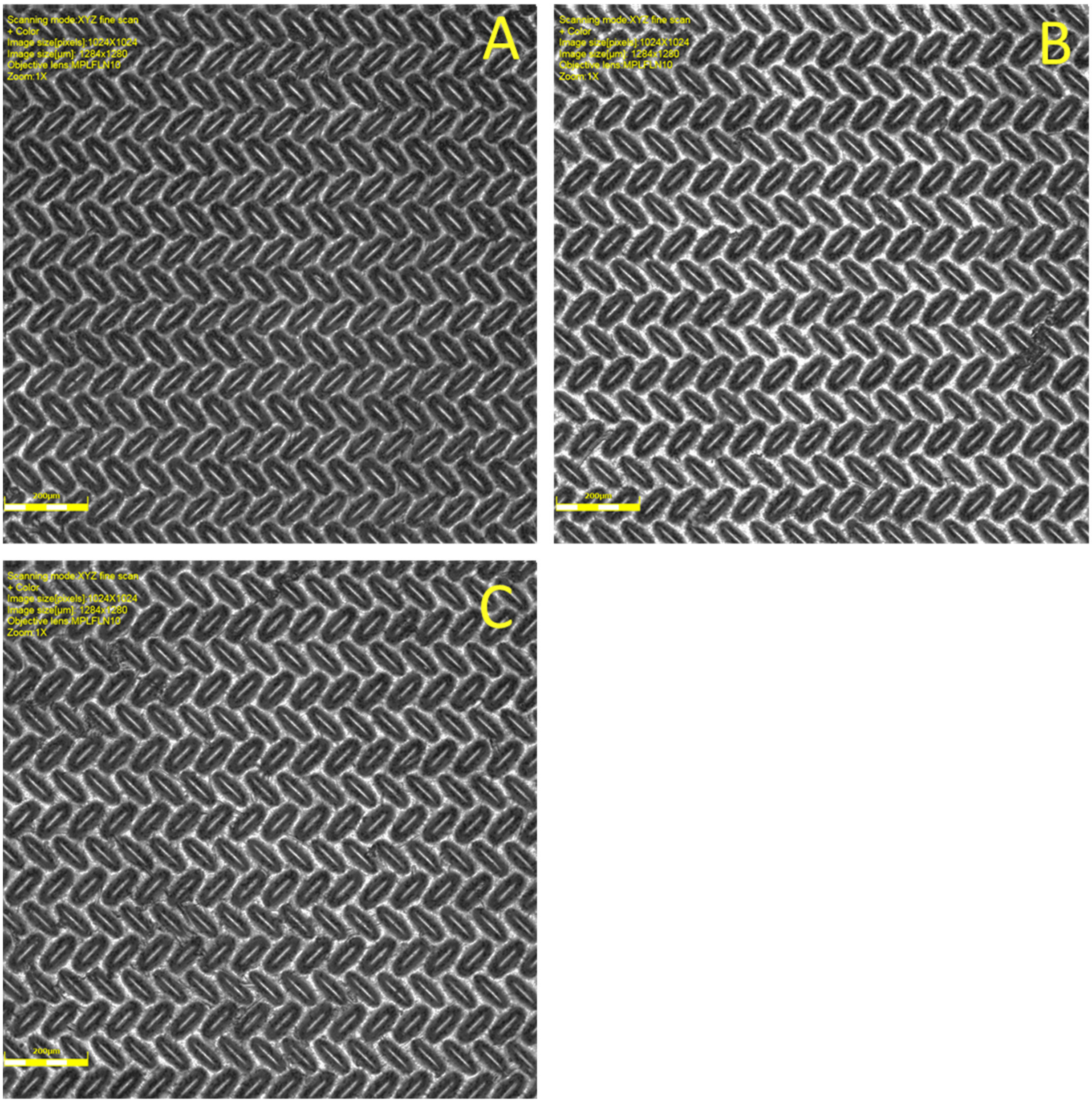

Figure S4. LEXT images of membranes patterned with same mesh stamp for (**A**) first time, (**B**) fifth time, (**C**) tenth time.

Hermia Model

To analyze the filtrate flux data, we replotted the flux decline results on a logarithmic scale of d^2t/dV^2 versus dt/dV as obtained from the Hermia model equation (Eq. [1]). As Ho and Zydney [41] described, the required derivatives were evaluated in terms of the filtrate flux.

$$\frac{dt}{dV} = \frac{1}{JA} \tag{1}$$

$$\frac{d^2t}{dV^2} = \frac{1}{J^3A^2}\frac{dJ}{dt} \tag{2}$$

dJ/dt was evaluated numerically by differentiating cubic polynomials that were fit to the flux versus time data of Figure 6. The n parameter of the Hermia model was obtained as the slope on the log–log plot of d^2t/dV^2 versus dt/dV:

$$n = \frac{d\left[\log\left(\frac{d^2t}{dV^2}\right)\right]}{d\left[\log\left(\frac{dt}{dV}\right)\right]} \quad (3)$$

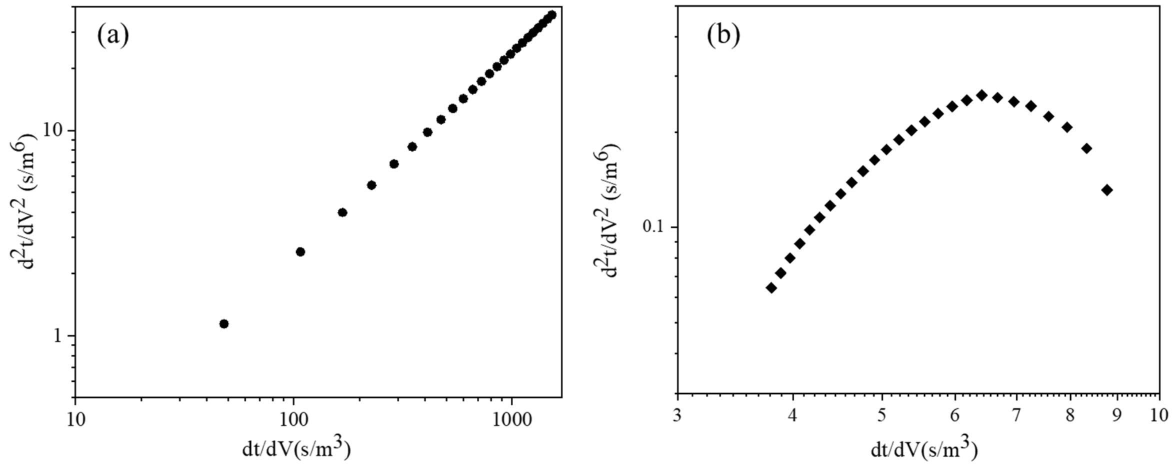

Figure S5. Flux decline analysis for bovine serum albumin (BSA) filtration with (**a**) patterned and (**b**) as-received membrane.

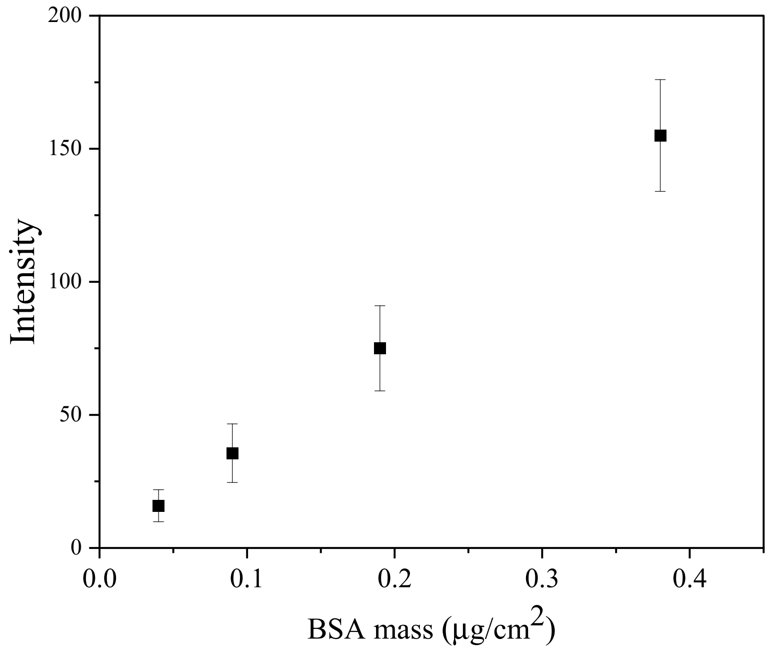

Figure S6. Calibration data relating fluorescence intensity to areal mass for BSA-Alexa Fluor™ 647 conjugate. The errors bars provide the corresponding standard deviation values.

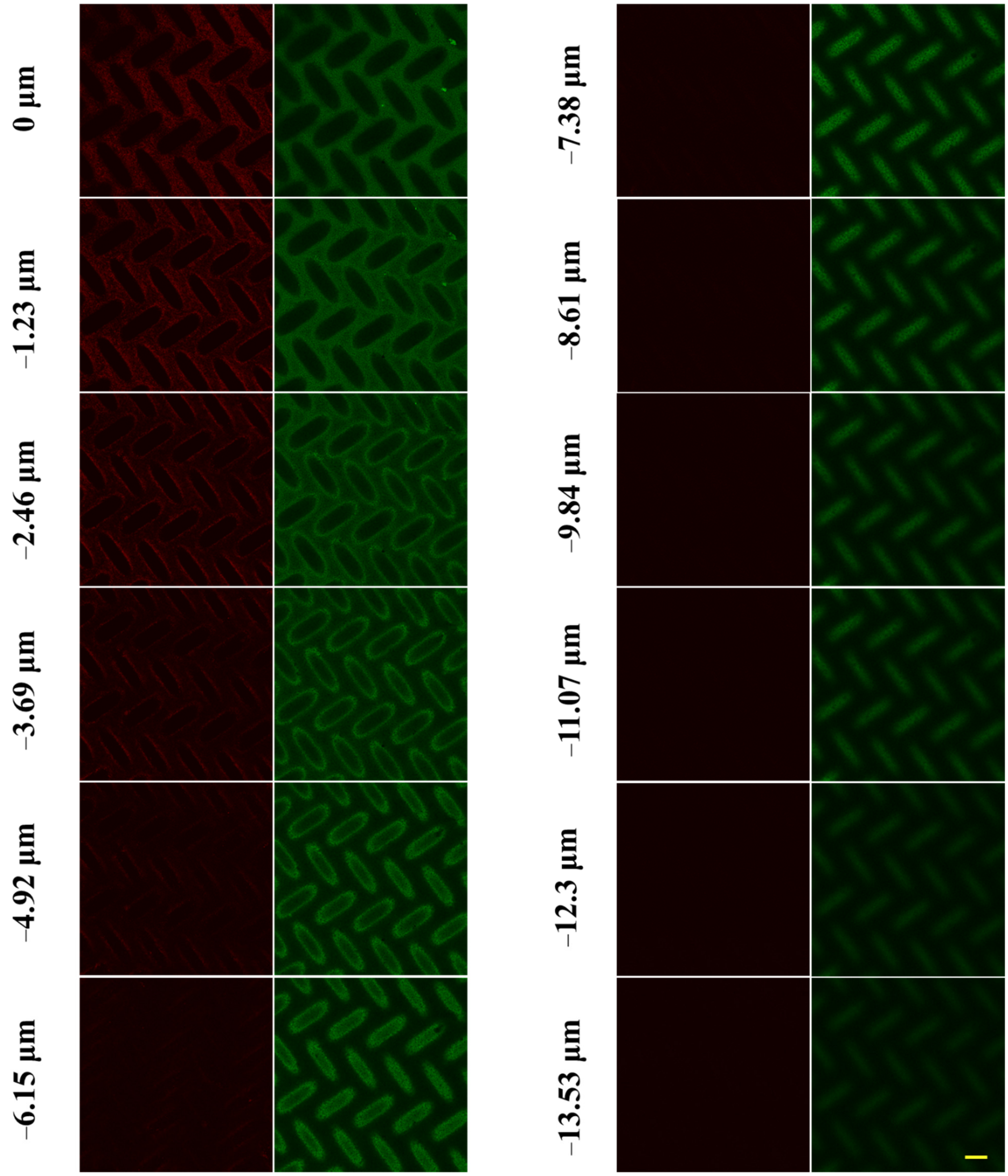

Figure S7. 3D orthogonal reconstruction of confocal laser scanning microscopy (CLSM) images: membrane after 10 s filtration of BSA solution. Images are shown for patterned membrane (green) and BSA foulant (red) as a function of depth from the membrane surface (Z = 0). For better observation by the reader, light corrections (Brightness: +40% and Contrast: −40%) were applied to the images. The common scale bar is 50 μm.

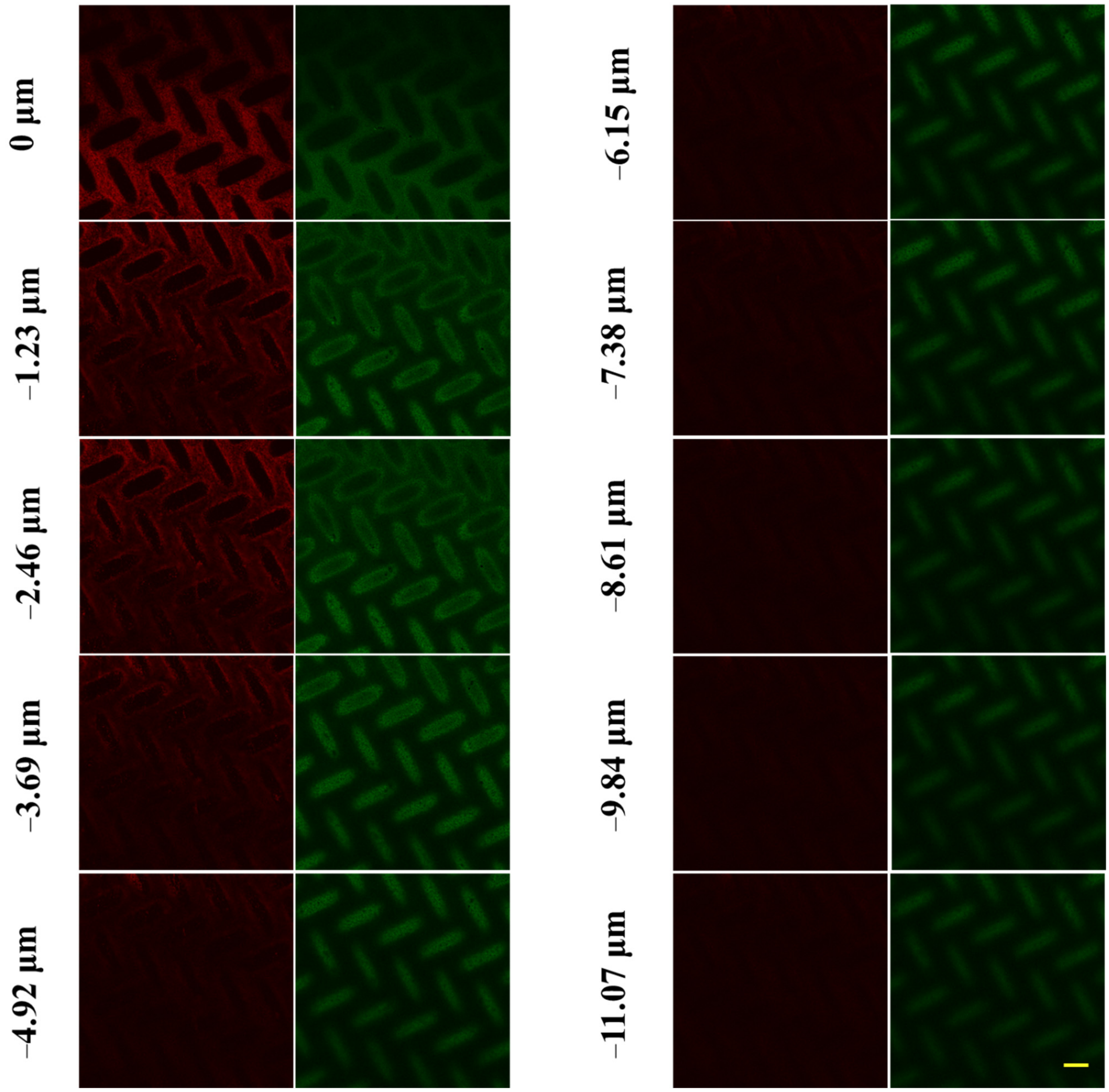

Figure S8. 3D orthogonal reconstruction of CLSM images: membrane after 20 s filtration of BSA solution. Images are shown for patterned membrane (green) and BSA foulant (red) as a function of depth from the membrane surface (Z = 0). For better observation by the reader, light corrections (Brightness: +40% and Contrast: −40%) were applied to the images. The common scale bar is 50 µm.

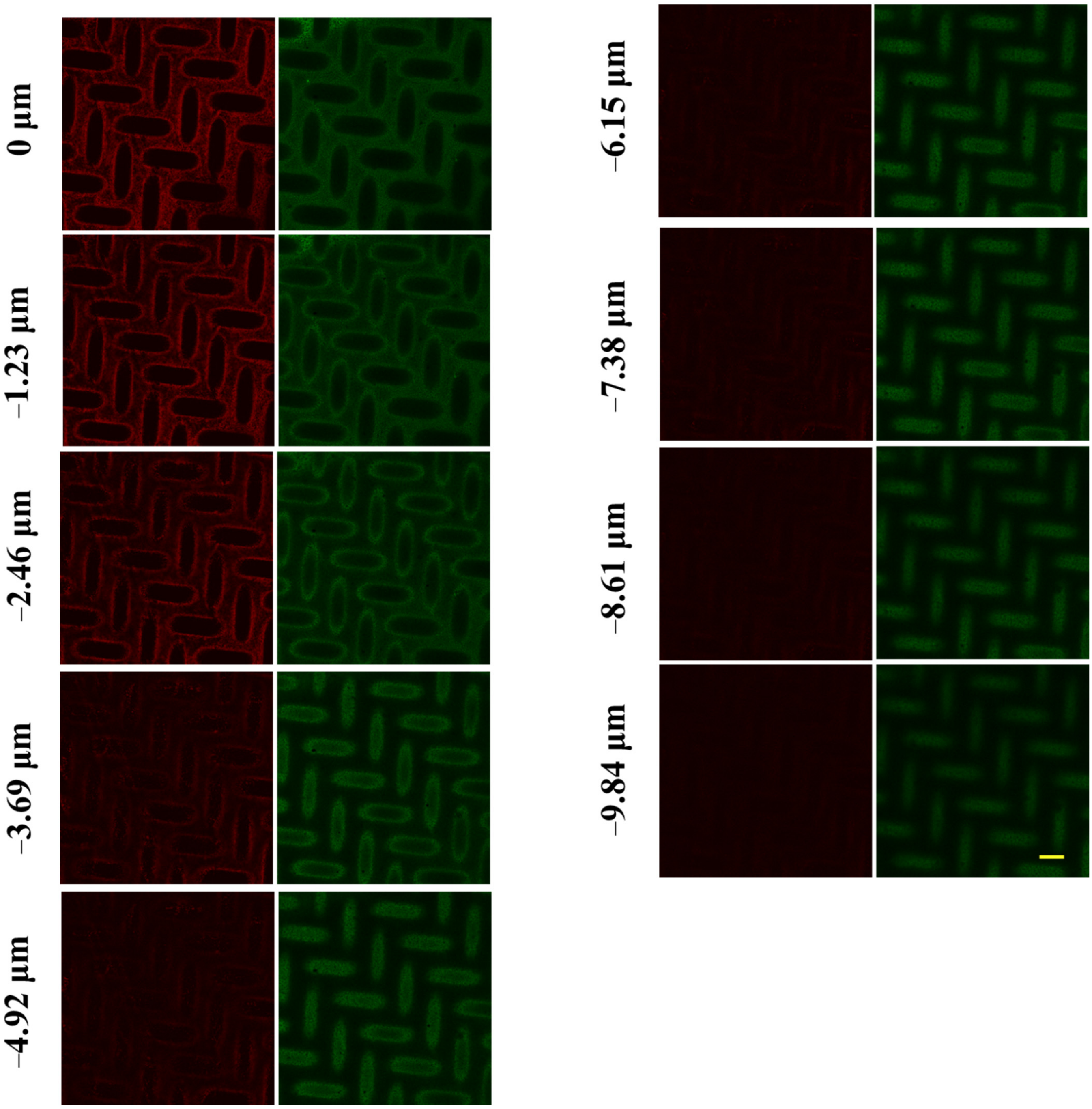

Figure S9. 3D orthogonal reconstruction of CLSM images: membrane after 30 s filtration of BSA solution. Images are shown for patterned membrane (green) and BSA foulant (red) as a function of depth from the membrane surface (Z = 0). For better observation by the reader, light corrections (Brightness: +40% and Contrast: −40%) were applied to the images. The common scale bar is 50 µm.

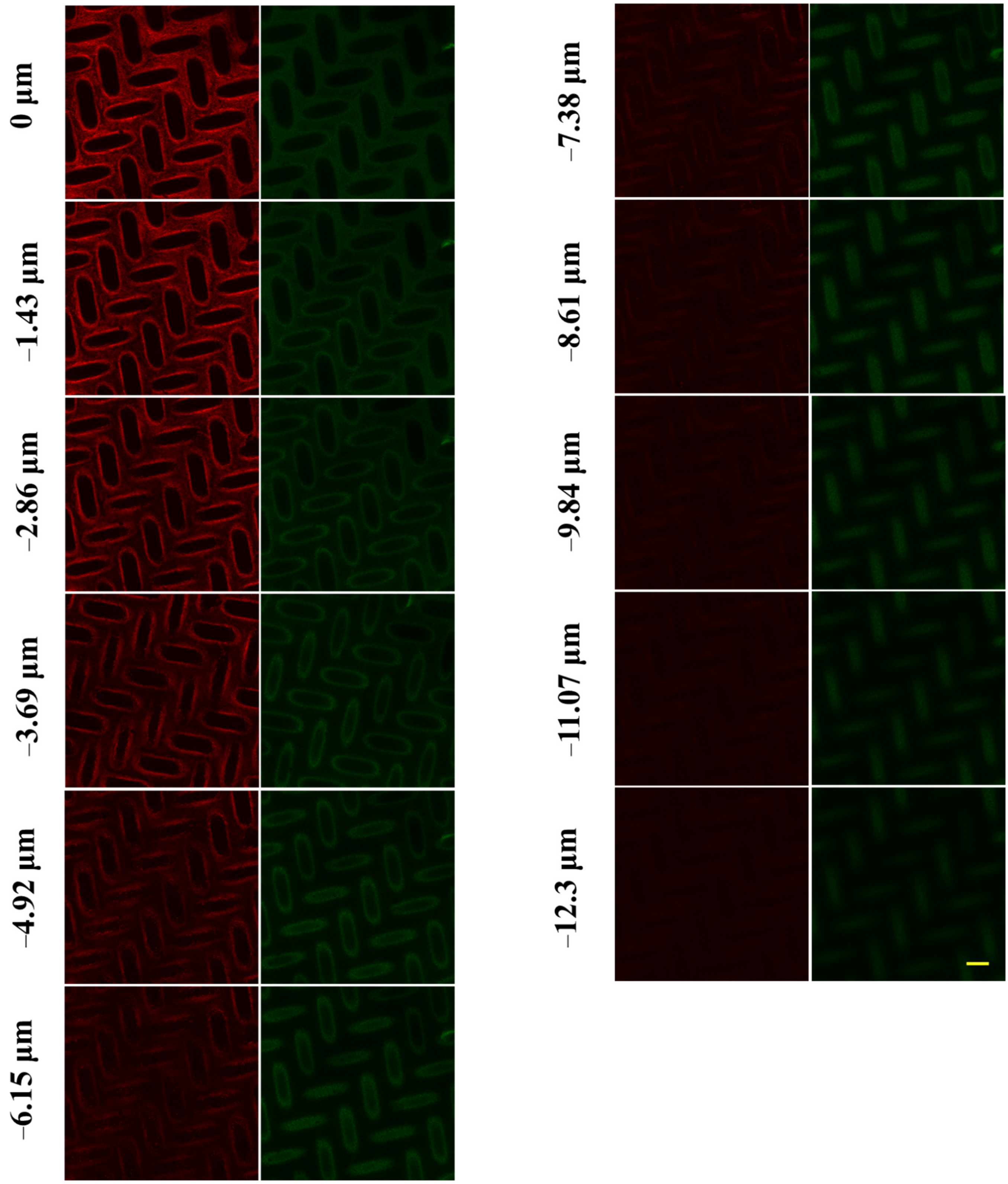

Figure S10. 3D orthogonal reconstruction of CLSM images: membrane after 60 s filtration of BSA solution. Images are shown for patterned membrane (green) and BSA foulant (red) as a function of depth from the membrane surface (Z = 0). For better observation by the reader, light corrections (Brightness: +40% and Contrast: −40%) were applied to the images. The common scale bar is 50 μm.

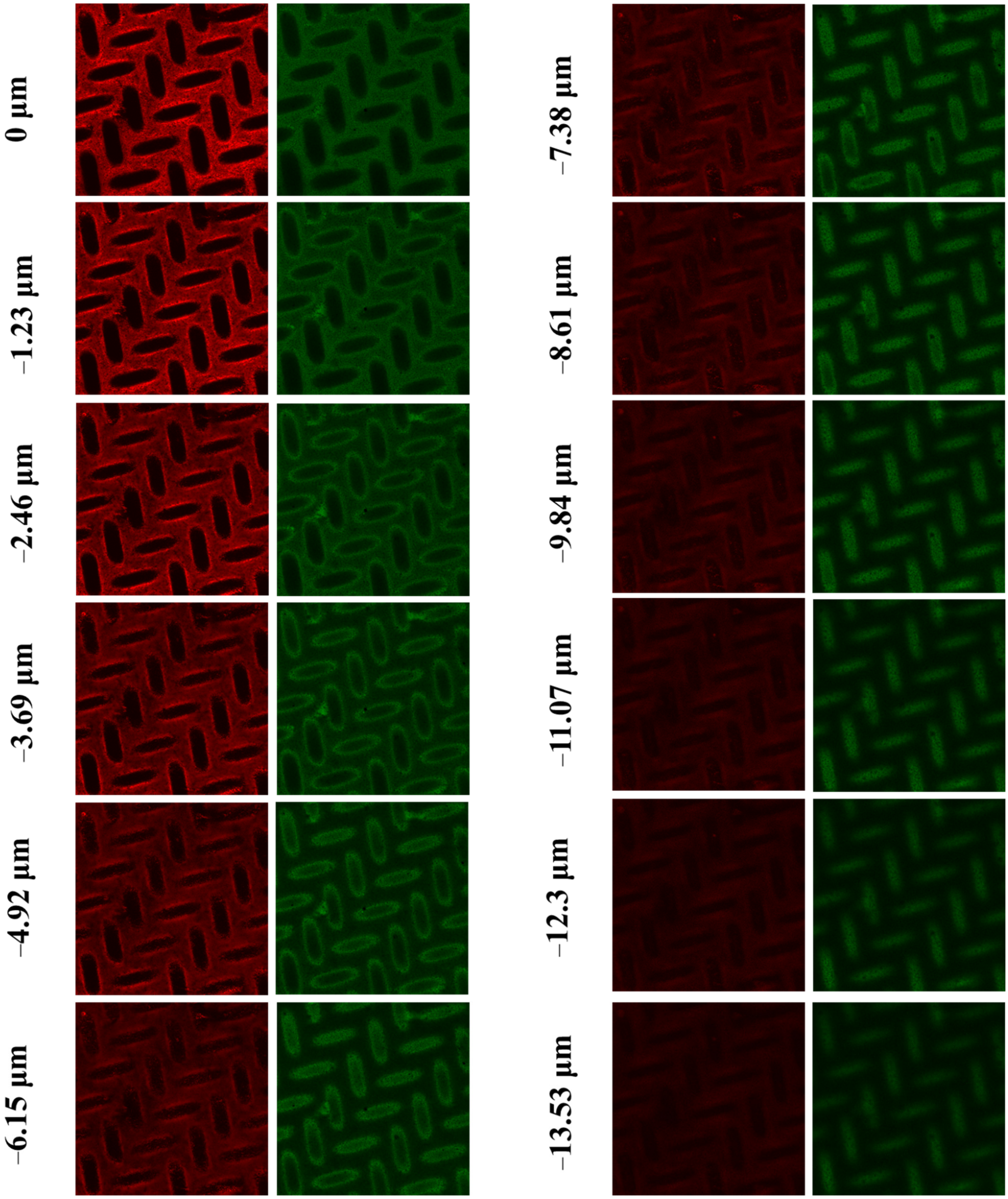

Figure S11. 3D orthogonal reconstruction of CLSM images: membrane after 5 min filtration of BSA solution. Images are Scheme 0. and Contrast: −40%) were applied to the images. The common scale bar is 50 μm.

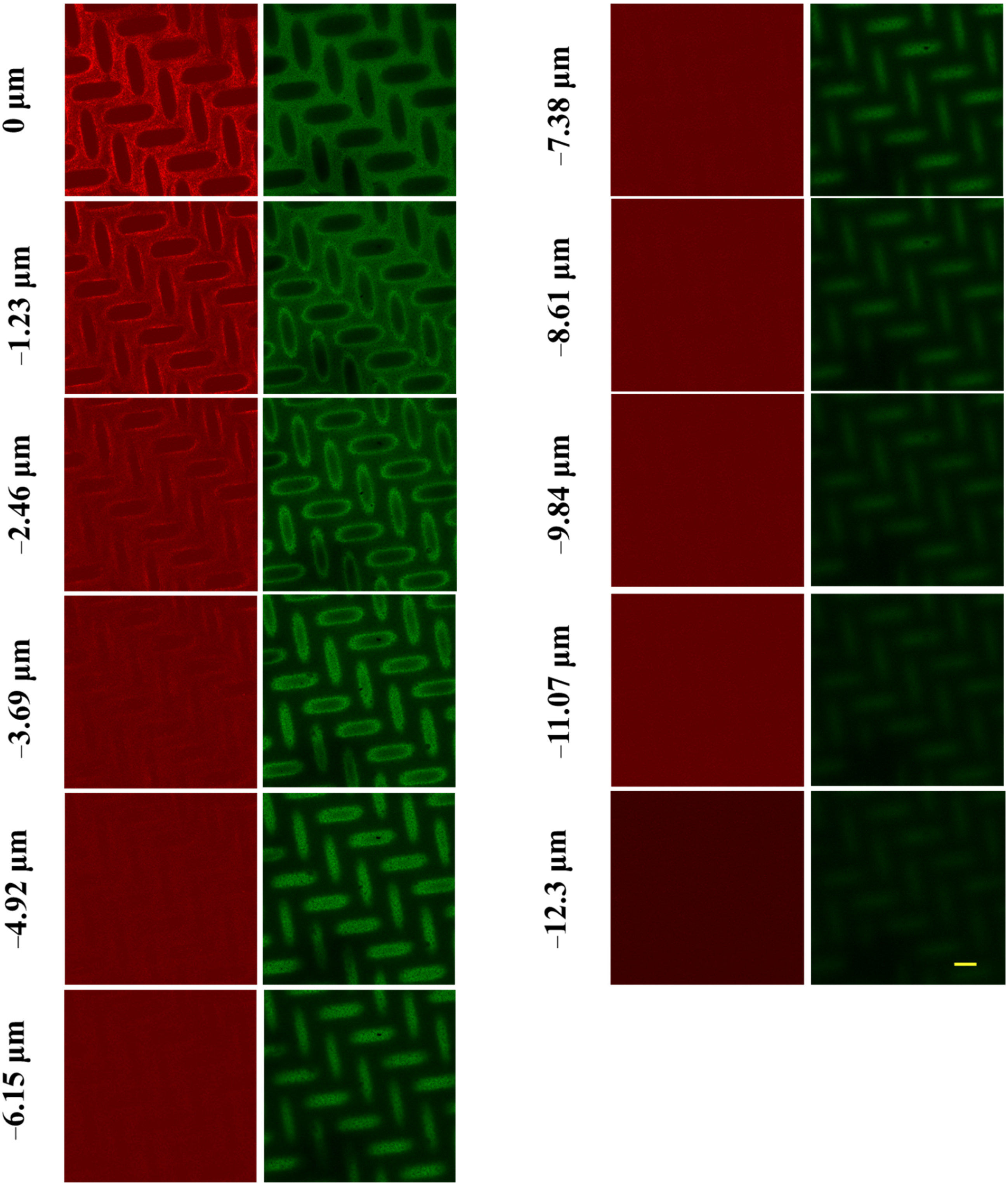

Figure S12. 3D orthogonal reconstruction of CLSM images: membrane after 10 min filtration of BSA solution. Images are shown for patterned membrane (green) and BSA foulant (red) as a function of depth from the membrane surface (Z = 0). For better observation by the reader, light corrections (Brightness: +40% and Contrast: −40%) were applied to the images. The common scale bar is 50 µm

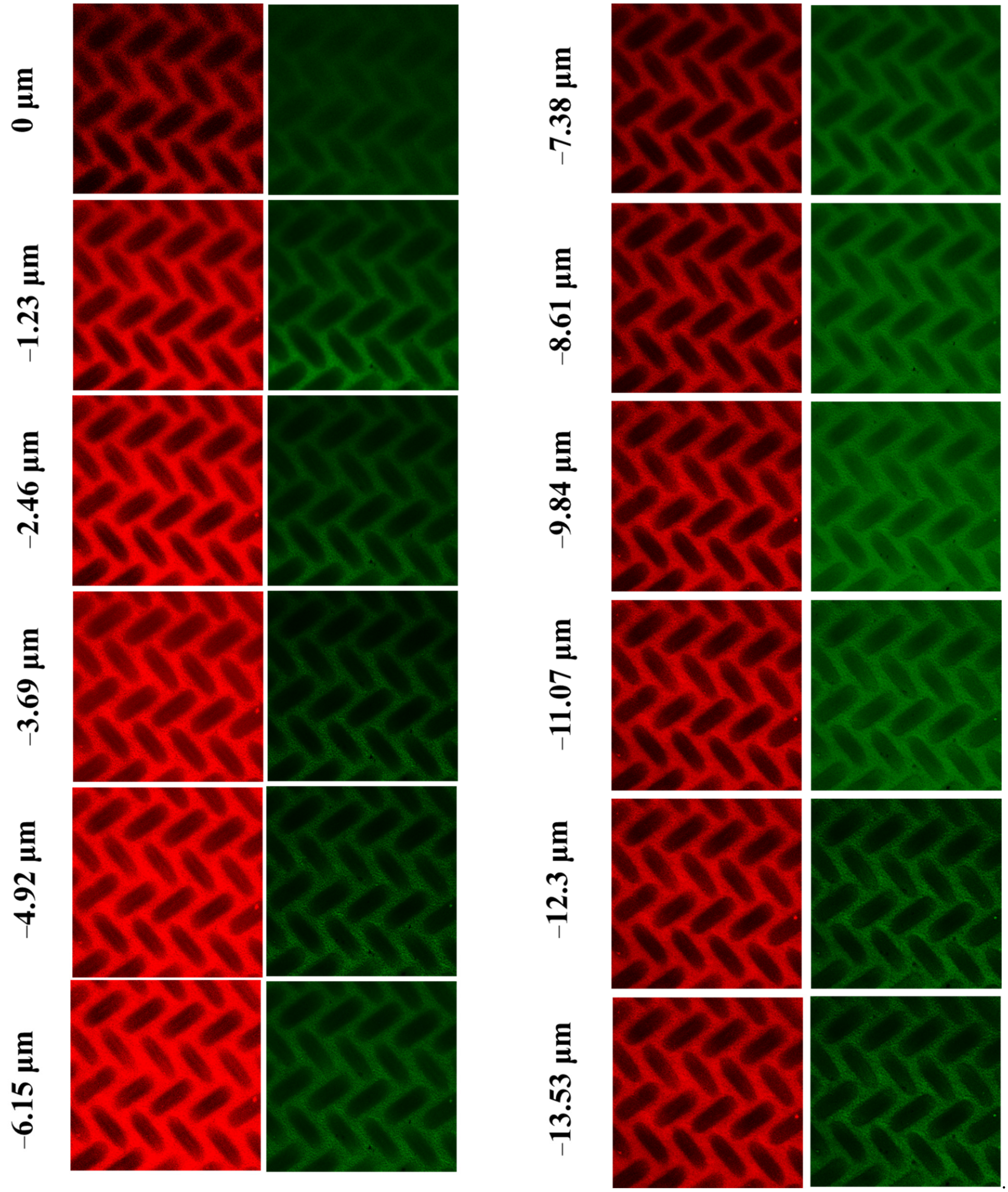

Figure S13. 3D orthogonal reconstruction of CLSM images: membrane after 30 min filtration of BSA solution. Images are shown for patterned membrane (green) and BSA foulant (red) as a function of depth from the membrane surface (Z = 0). For better observation by the reader, light corrections (Brightness: +40% and Contrast: −40%) were applied to the images. The common scale bar is 50 µm.

Results of Statistical Tests

Table S1. Results of paired t-tests on average membrane pattern feature sizes comparing the first and tenth use of the same woven mesh as a stamp.

Measured Value	Two-tailed P value	Result
Depth	0.007	**No significant difference** *
Width	0.019	**No significant difference**
Length	< 0.001	**No significant difference**

*Indicates there is strong evidence that the null hypothesis is incorrect (i.e., mean values are the same).
